# Early Transcriptional Landscapes of *Chlamydia trachomatis*-Infected Epithelial Cells at Single Cell Resolution

**DOI:** 10.3389/fcimb.2019.00392

**Published:** 2019-11-19

**Authors:** Regan J. Hayward, James W. Marsh, Michael S. Humphrys, Wilhelmina M. Huston, Garry S. A. Myers

**Affiliations:** ^1^Faculty of Science, School of Life Sciences, The ithree Institute, University of Technology Sydney, Ultimo, NSW, Australia; ^2^Department of Microbiome Science, Max Planck Institute for Developmental Biology, Tübingen, Germany; ^3^Institute for Genome Sciences, University of Maryland School of Medicine, Baltimore, MD, United States; ^4^Faculty of Science, School of Life Sciences, University of Technology Sydney, Ultimo, NSW, Australia

**Keywords:** *Chlamydia* (*Chlamydia trachomatis*), infection, single cell, transcriptomics, bioinformatics

## Abstract

*Chlamydia* are Gram-negative obligate intracellular bacterial pathogens responsible for a variety of disease in humans and animals worldwide. *Chlamydia trachomatis* causes trachoma in disadvantaged populations, and is the most common bacterial sexually transmitted infection in humans, causing reproductive tract disease. Antibiotic therapy successfully treats diagnosed chlamydial infections, however asymptomatic infections are common. High-throughput transcriptomic approaches have explored chlamydial gene expression and infected host cell gene expression. However, these were performed on large cell populations, averaging gene expression profiles across all cells sampled and potentially obscuring biologically relevant subsets of cells. We generated a pilot dataset, applying single cell RNA-Seq (scRNA-Seq) to *C. trachomatis* infected and mock-infected epithelial cells to assess the utility, pitfalls and challenges of single cell approaches applied to chlamydial biology, and to potentially identify early host cell biomarkers of chlamydial infection. Two hundred sixty-four time-matched *C. trachomatis*-infected and mock-infected HEp-2 cells were collected and subjected to scRNA-Seq. After quality control, 200 cells were retained for analysis. Two distinct clusters distinguished 3-h cells from 6- and 12-h. Pseudotime analysis identified a possible infection-specific cellular trajectory for *Chlamydia*-infected cells, while differential expression analyses found temporal expression of metallothioneins and genes involved with cell cycle regulation, innate immune responses, cytoskeletal components, lipid biosynthesis and cellular stress. We find that changes to the host cell transcriptome at early times of *C. trachomatis* infection are readily discernible by scRNA-Seq, supporting the utility of single cell approaches to identify host cell biomarkers of chlamydial infection, and to further deconvolute the complex host response to infection.

## Introduction

*Chlamydia* are Gram-negative obligate intracellular bacterial pathogens that cause disease in humans and a wide variety of animals. In humans, *Chlamydia trachomatis* typically infects cells within the ocular and genital mucosa, causing the most prevalent bacterial sexually transmitted infections (STI; Reyburn, 2016), inducing acute and chronic reproductive tract diseases that impact all socioeconomic groups, and trachoma in disadvantaged populations (Burton and Mabey, 2009). Disease outcomes arise from complex inflammatory cascades and immune-mediated host processes that can lead to tissue damage and fibrotic scarring in the upper genital tract or the conjunctiva (Taylor et al., 2014; Menon et al., 2015). Reproductive tract disease outcomes include pelvic inflammatory disease (PID), preterm delivery, ectopic pregnancy, hydrosalpinx, tubal factor infertility (TFI), and chronic pelvic pain in women, as well as epididymitis, testicular pain and infertility in men. Antibiotic therapy with azithromycin or doxycycline successfully treats diagnosed infections, however asymptomatic infections are common (Hafner et al., 2014; Ali et al., 2015). Without overt symptoms that lead individuals to seek primary health care, antibiotic interventions are not able to be employed. Asymptomatic infection rates are estimated to exceed diagnosed infection rates by at least 4.3-fold (Ali et al., 2015).

*Chlamydia* have a unique biphasic developmental cycle with distinct morphological forms. The cycle begins with attachment and entry of the infectious elementary bodies (EBs) into host cells, typically mucosal epithelial cells. After entry, EBs reside within membrane-bound vacuoles that escape phagolysomal fusion (Scidmore et al., 1996). Differentiation into the replicating reticulate bodies (RBs) occurs within the first 2–3 h, followed by continued growth of the inclusion accommodating the increased number of RBs. Over the course of infection, *Chlamydia* parasitises and modifies the host cell by deploying type III effectors and other secreted proteins (Valdivia, 2008), which also facilitate invasion, internalization, and replication, while countering host cell defenses (Saka et al., 2011; Bastidas et al., 2013). At the end of the developmental cycle, RBs asynchronously transition back into EBs (~20–44 h) and, through either extrusion or host cell lysis (~48–70 h), are released to repeat the cycle (Elwell et al., 2016).

Chlamydial transcriptomes have been examined over the developmental cycle, in EBs and RBs, in different chlamydial species (Belland et al., 2003; Albrecht et al., 2010, 2011; Abdelrahman et al., 2011). Epithelial cell transcriptomes responding to plasmid-bearing/plasmid-less *C. trachomatis* has been characterized by microarray (Porcella et al., 2015). Dual RNA-Seq (Humphrys et al., 2013; Marsh et al., 2018) has allowed the transcriptomes of both *C. trachomatis* and infected epithelial cells to be profiled simultaneously, identifying previously unrecognized early chlamydial gene expression and complex host cell responses (Humphrys et al., 2013). However, in these studies to date, the derived transcriptional profiles represent averaged gene expression over the population of cells sampled (Hebenstreit, 2012). Subsets of cells with dominant gene expression profiles can skew the analysis (Łabaj et al., 2011), possibly obscuring other potentially important cell subsets and their transcriptional profiles (Saliba et al., 2014; Liu and Trapnell, 2016). By examining the expression profiles of individual cells, single cell RNA sequencing (scRNA-Seq) can minimize these biases, enabling a deeper understanding of population heterogeneity, cell states and interactions, and gene regulation (Kolodziejczyk et al., 2015; Regev et al., 2017). scRNA-Seq and other single cell methods have been instrumental in discovering new cell types (Regev et al., 2017) and advancing the understanding of many disease states (Sandberg, 2013), particularly tumor heterogeneity (Patel et al., 2014; Tirosh et al., 2016), hematopoiesis (Kowalczyk et al., 2015), and embryonic development (Yan et al., 2013). Applications of scRNA-Seq to pathogen-infected cells are more limited so far, but are exemplified by studies that show the heterogeneity of macrophage responses to *Salmonella enterica* serovar Typhimurium infection (Saliba et al., 2016), the high degree of cell-cell transcriptional variation induced by influenza virus infection (Russell et al., 2018), and the characterization of lymph node-derived innate responses to bacterial, helminth, and fungal pathogens (Blecher-Gonen et al., 2019).

Here we explore the application of single cell analysis methodologies to *Chlamydia*-infected cells, with the goals of identifying host cell developmental-stage biomarkers, and to assess the utility of these methodologies for deciphering chlamydial biology in cells and tissues. We generated a pilot scRNA-Seq dataset of time-matched infected and mock-infected HEp-2 epithelial cells *in vitro* encompassing the early chlamydial developmental cycle (3, 6, and 12 h). We show that infection responsive changes to the early host cell transcriptome are readily discernible by scRNA-Seq, supporting the potential for host derived infection biomarkers.

## Results

### Single Cell Capture, Library Construction, Quality Assessment, and Filtering

**Figure 1:**
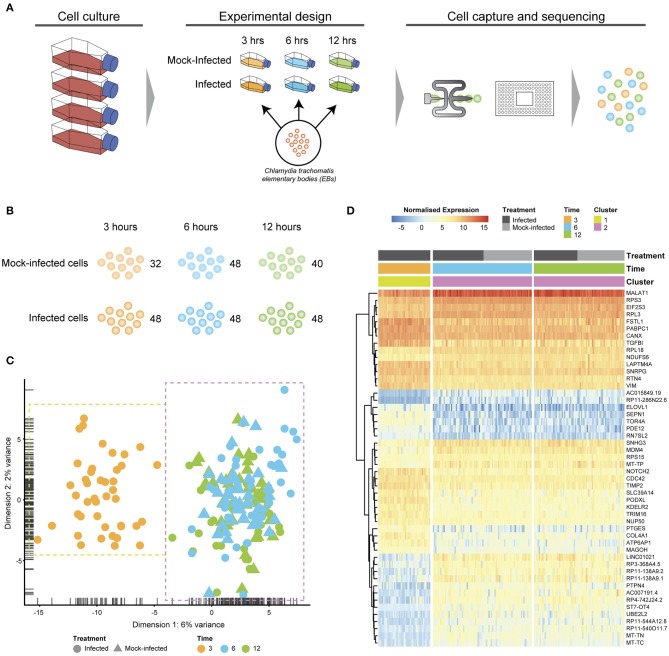
Experimental design and analysis. **(A)** Cell culture using HEp2 epithelial cell monolayers used to grow and harvest *Chlamydia trachomatis* E elementary bodies (EBs). Fresh monolayers were infected with EBs, (MOI ~1) using centrifugation to synchronize infections. Experimental design time-matched *Chlamydia*-infected and mock-infected cell monolayers at 3, 6 and 12 h, prior to capture and scRNA-Seq library preparation on the Fluidigm C1 instrument. **(B)** Numbers of captured and sequenced single cells by experimental condition and time. **(C)** After quality control steps, unsupervised clustering identifies two primary clusters. Cluster 1 contains all 3 h cells, while cluster 2 contains all 6 and 12 h cells. **(D)** Putative marker genes grouped by hierarchical clustering.

*Chlamydia*-infected (*C. trachomatis* serovar E, MOI~1) and time-matched mock-infected cells spanning three times post-infection were captured using the Fluidigm C1 microfluidic instrument and workflows (Figure 1A). We obtained 80 single cells at 3 h (48 *Chlamydia*-infected, 32 mock-infected), 96 cells at 6 h (48 *Chlamydia*-infected, 48 mock-infected), and 88 cells at 12 h (40 *Chlamydia*-infected, 48 mock-infected) for an initial total of 264 cells (Figure 1B). Following Illumina library construction and sequencing, the raw sequencing reads from these 264 cells were demultiplexed using DeML (Renaud et al., 2015), yielding 1.03 billion sequence reads (Supplemental File 1). Single cell datasets were removed from subsequent analyses if they contained <1 million reads after trimming and alignment, and <5,000 counted features (genes). Further quality assessment measures ensured that sequence reads mapped across all chromosomes and that the majority of reads mapped to protein-coding genes (Supplemental File 2). Single cell datasets were pooled and subjected to additional quality assessment steps, including examining rRNA as a measure of depletion success and mitochondrial gene expression as an indicator of cell stress (Zhao et al., 2002), as both are potential sources of bias (Supplemental File 3). During quality control, the mock-infected cells at 3 h failed to pass cut-offs, and were excluded from further downstream analysis (Supplemental File 3). After all quality measures, datasets from 200 high quality single cells remained across the three times: 43 *Chlamydia*-infected cells at 3 h; 82.6 h cells (42 *Chlamydia*-infected, 40 mock-infected), and 75.12 h cells (36 *Chlamydia*-infected, 39 mock-infected).

### Removal of Confounding Effects

To normalize by library size, Scran's single-cell specific method was used to deconvolute library size factors from cell clusters (Lun et al., 2016). We applied RUVSeq (Risso et al., 2014) to identify and remove further confounding effects, including differences between batches of sequenced cells. Reduction of variation was confirmed in relative log expression (RLE) plots (Supplemental File 4A). Density curve plots further show the effect of removing variability from the raw counts, after library size normalization, and after removing further confounding effects (Supplemental File 4B). The PCA bi-plot (Supplemental File 4C) shows the structure of the data and grouping of the cells based on their transcriptional profiles following these steps. By examining the underlying variables driving PC1 variation, we found that total read counts and time post-infection account for 99% of the total variation (Supplemental File 4C), confirming that most variation is not from experimental factors. In addition, doublets (where at least two cells are captured into the same well) can skew the resulting expression profiles, adding a further confounding factor. Although the C1 platform uses integrated fluidic circuits (IFCs) to isolate single cells, it has been associated with a doublet rate as high as 25% (Wang et al., 2019). Due to this high reported rate, we ran different tools to identify doublets, confirming that our data had minimal detected doublets (Supplemental File 5).

### Cell Cycle Classification

**Figure 2:**
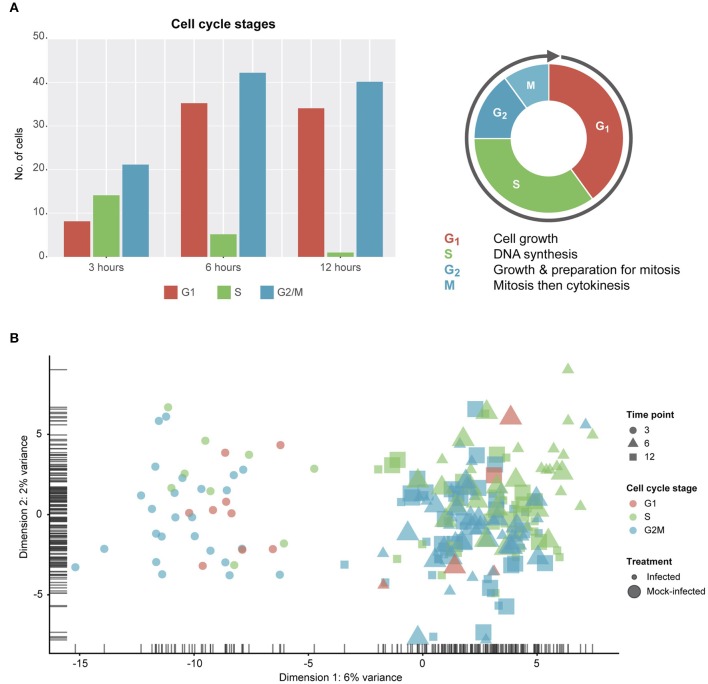
Cell cycle classification. **(A)** Cell cycle classification of single cells after removing outliers. **(B)** PCA plot examining cell-cycle related trends by time-point and infection status.

Due to the constraints imposed by chlamydial infection within *in vitro* tissue culture and, given the potential for cell-cell variability despite infection synchronization, we expected to observe a range of cell cycle stages in our data (Figure 2). Two of the three stages (G1 and G2/M) show more than double the number of cells from 3 to 6 h, while DNA synthesis (S) is the only cell cycle stage with a decrease in the number of cells from 3 to 12 h. However, despite these trends, no distinct cell cycle clusters are apparent (Figure 2B). In addition, there is no clustering between cell cycle state and time post-infection, or infection condition (infected vs. mock-infected). Although we identify cell cycle stage as a likely confounding effect (Barron and Li, 2016) that was removed from our subsequent analyses, it may be relevant to the infection and growth strategies of *Chlamydia*. For example, while infected cells can still grow and divide, the burden of infection causes these cells to proliferate more slowly than uninfected cells, resulting in dividing cells which may be more or less susceptible to infection (Balsara et al., 2006). Additionally, chlamydial infectivity has been related to distinct cell cycle phases, where infection can modulate cell cycle parameters (Johnson et al., 2009).

### Clustering Demonstrates Transcriptional Heterogeneity of Infected Epithelial Cells Over the Early Chlamydial Developmental Cycle

**Figure 3:**
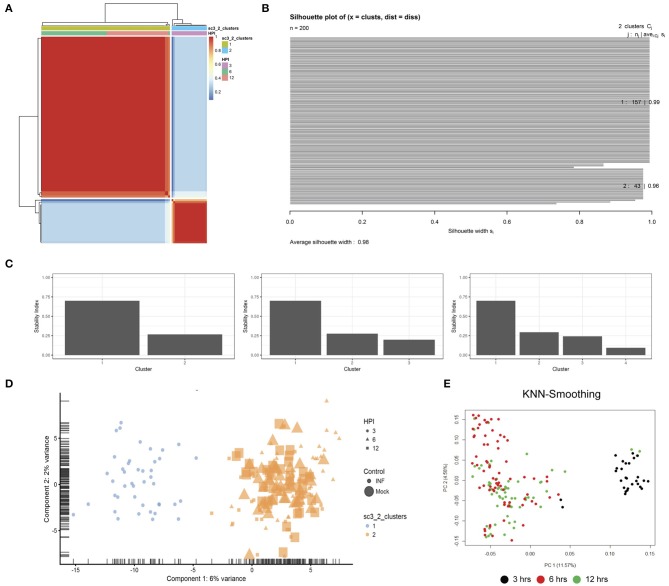
Clustering. **(A)** SC3 consensus matrix predicted 2 clusters, dark red coloring. **(B)** Silhouette plot of the consensus matrix (100% indicates perfect clustering). **(C)** Cluster stability plots showing that as the number of clusters increases past two, cluster stability decreases. **(D)** PCA plot of the two predicted clusters, colored by time-point, sized by infection status and shaped by cluster. **(E)** PCA plot following kNN-smoothing on the expression matrix.

**Figure 4:**
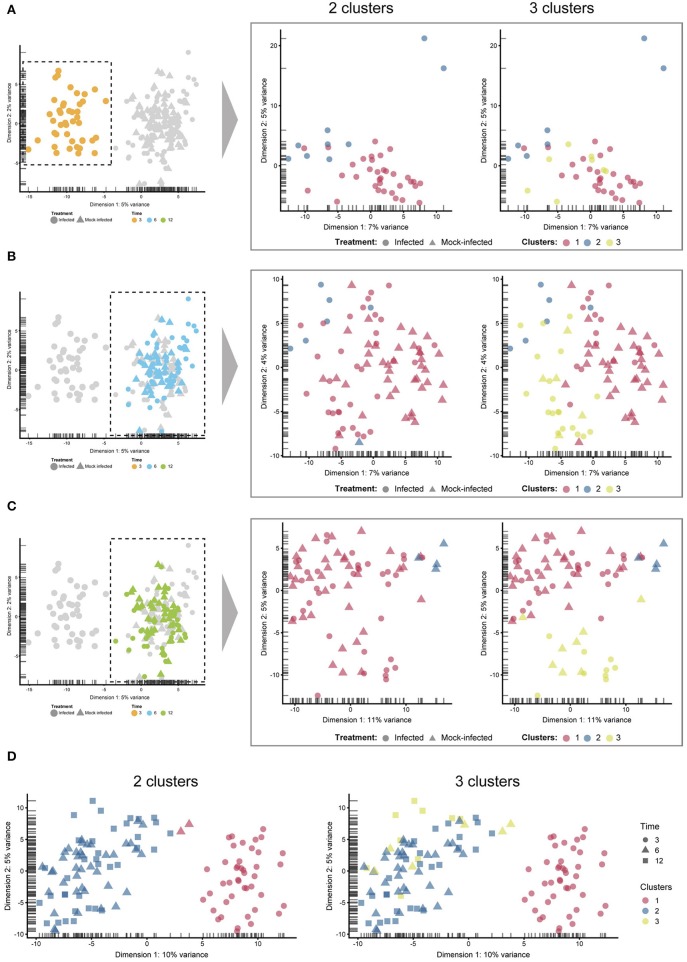
Sub-clustering. The four comparisons shown here were created by manually selecting two and three clusters to examine any sub-clustering events not automatically detected. **(A)** 3 h cells—no sub-clustering evident. **(B)** 6 h cells—no apparent sub-clustering with two clusters; three clusters do display more of a separation (between blue and green), while infected and mock-infected cells cannot be distinguished. **(C)** 12 h cells—some separation evident with 3 clusters, but infection state is not distinguishable. **(D)** Extracting only infected cells show a clear separation of 3 h cells, but not 6 and 12 h cells.

Unsupervised clustering identified two distinct clusters across the three time points (Figures 1C, 3). Cluster 1 contains only 3-h infected cells, while cluster 2 contains a mixture of cells from 6 and 12 h, with no clear separation between infected and uninfected conditions. We used k-nearest neighbor smoothing (kNN-smoothing) to further reduce scRNA-Seq-specific noise within the expression matrix (Wagner et al., 2018), which is a common occurrence from effects such as dropouts (Gong et al., 2018). The resulting PCA plot recapitulated the clusters identified above, indicating that the previous clustering result was not influenced by noise-related factors. Additional clustering analyses were performed to identify any sub-populations within each cluster on the basis of experimental factors such as time or infection status (Figure 4). *Chlamydia*-infected cells again clustered into two main groups, closely matching the overall clustering that separated the 3 h cells from 6 and 12 h, with no further sub-clustering evident.

### Pseudotime Analysis Over the Early Chlamydial Developmental Cycle

**Figure 5:**
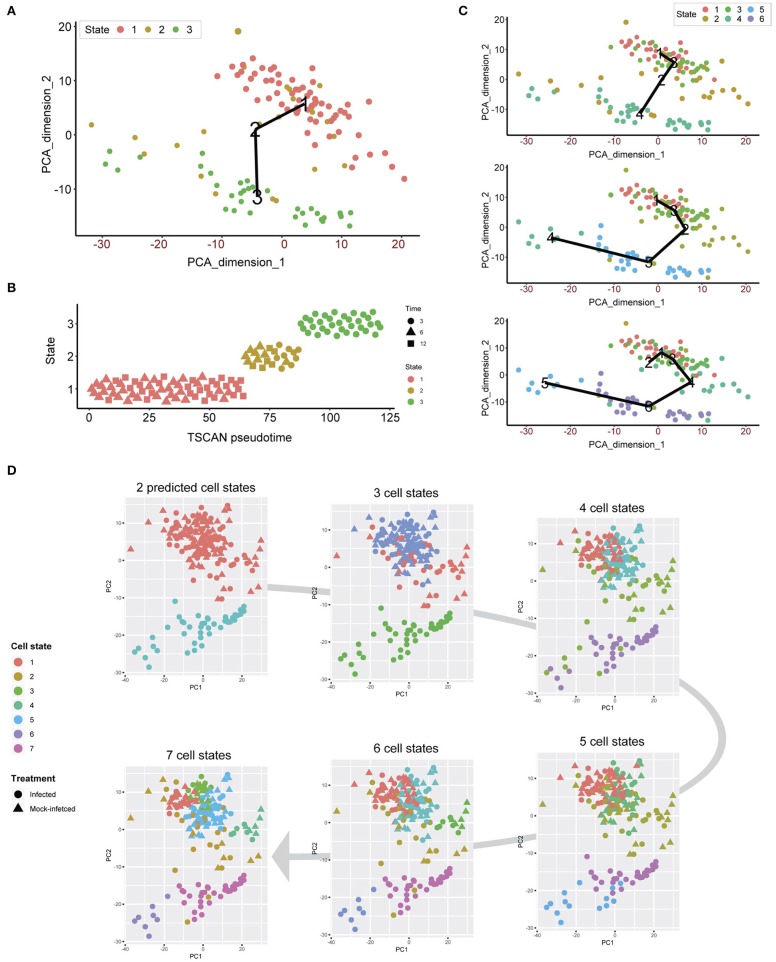
Pseudotime analysis. **(A)** Pseudotime analysis of infected cells predicts three cell states. The minimum spanning tree (black line) is uninformative and not a true indication of an expected infection trajectory encompassing all cells from 3 to 12 h. **(B)** Each cell ordered throughout the predicted pseudotime and separated by cell state. **(C)** Manually increasing the number of cell states to six appears to show a more realistic infection trajectory with a wider number of cells, in addition to showing start and end points correlating to 3 and 12 h cells. **(D)** When all cells are used, two cell states are predicted that support the initial clustering outcomes. When the number of cell states is manually increased, smaller subsets appear, providing a finer resolution.

Unsupervised clustering demonstrates that both infected and uninfected cells have minimal cluster separation at 6 and 12 h (Figure 1C). We applied pseudotime analysis to further deconvolute cellular trajectories that may follow a time course or biological mechanisms such as differentiation or infection (Ji and Ji, 2016; Lönnberg et al., 2017). Pseudotime analysis of *Chlamydia*-infected cells alone predicted 3 distinct cell states (Figure 5A). Cell state 1 contained 3 h cells, state 2 contained a mixture of 3, 6 and 12 h cells, and state 3 contains a mixture of 6 and 12 h cells (Figure 5B). The line connecting the 3 cell states (minimum spanning tree) does not provide a realistic linear trajectory of the infection course from 3 to 12 h. Manually increasing the number of states does provide a more realistic trajectory (Figure 5C); however, the similarity of 6 and 12 h cells (both mock-infected and infected states) will require more cells to accurately capture any putative sub-stages of infection.

We overlaid the predicted cell cycle states from pseudotime analysis for each cell to identify any shared characteristics of infected cells with mock-infected cells, which could classify cells that were either not infected or had unproductive infections. This analysis identified only two cell states (Figure 5D), recapitulating the initial clustering results. By manually increasing the number of cell states to 7, smaller sub-clusters within the two main clusters became evident. However, we still observe a mixture of infected and mock-infected cells within each sub-cluster (albeit with small numbers of cells), further highlighting the transcriptional similarity between infected cells at 6 and 12 h in this dataset.

### Differentially Expressed Genes in *Chlamydia*-Infected and Mock-Infected Cells Highlight Infection Mechanisms

Subsets of genes with significant expression differences between the two primary clusters were examined in order to identify any putative host cell marker genes that distinguish different times post-infection (Figure 1D). At 6 and 12 h, genes associated with processes governing RNA and protein metabolism (*RPS3, RPL3, RPS15, RPL18, PABPC1, MAGOH*, and *EIF2S3*) predominate, with most showing increased expression. Increased expression of vimentin (*VIM*), a type III intermediate filament (IF) present in the cytoskeleton and involved in maintaining cell shape and integrity (Mak and Brüggemann, 2016), distinguishes the 3 h cluster.

**Figure 6:**
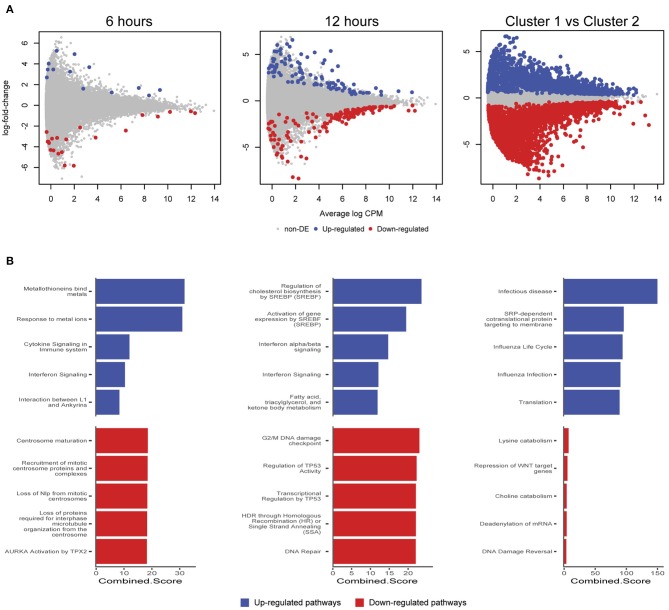
Differentially expressed genes and enriched pathways. **(A)** Differentially expressed genes from infected and mock-infected cells at 6 and 12 h. When comparing cells from cluster 1 against cluster 2, a more complex experimental design was needed that took into consideration the variety of underlying cells. **(B)** Enriched pathways from Reactome using differentially expressed genes from **(A)**.

We further examined differentially expressed (DE) genes firstly by comparing infected and mock-infected cells at 6 and 12 h, respectively (cluster 2), and secondly by comparing the 3 h infected cells (cluster 1) against cluster 2, as the 3 h mock-infected cells were removed after initial quality control steps. At 6 h, 44 DE genes were identified (13 up-regulated and 21 down-regulated; Figure 6A), including three up-regulated metallothionein (MT) genes (*MT1E, MT2A*, and *MT1X*). MT up-regulation occurs in response to intracellular zinc concentration increases, reactive oxygen species (ROS) and proinflammatory cytokines (Rice et al., 2016). Intracellular zinc concentrations are an integral component of immunity and inflammation, and zinc deficiency results in an increased susceptibility to infection (Subramanian Vignesh and Deepe, 2017). MTs may also have a role in protecting against DNA damage and in apoptosis, as well as regulating gene expression during the cell cycle (Cherian and Apostolova, 2000), which are likely to be relevant at 6 h post-infection. Down-regulated pathways at 6 h were dominated by three genes *HSP90AA1* (Heat Shock Protein 90 Alpha Family Class A Member 1), *TUBB* (Tubulin Beta Class I), and *TUBA4A* (Tubulin Alpha 4a), which are linked to the cell cycle, specifically centrosome maturation and microtubule assembly mediating mitosis (Figure 6B).

At 12 h, there is an increase in DE genes (245) with 98 up-regulated and 147 down-regulated (Figure 6A). We continue to see up-regulated genes that are likely part of a continued immune response to infection, including two MTs (*MT1M* and *MT1E*), *TRIM25* (Tripartite Motif Containing 25), *ISG15* (*ISG15* Ubiquitin Like Modifier), *HLA-A* (Major Histocompatibility Complex, Class II, DR Beta 1), *IFIT3* (Interferon Induced Protein With Tetratricopeptide Repeats 3), *OASL* (2′-5′-Oligoadenylate Synthetase Like), *IL6* (Interleukin 6), and genes associated with cholesterol and fatty acid synthesis (Figure 6B). The exploitation of a variety of host lipids by *Chlamydia* to subvert intracellular signaling, survival and growth is well established (Kumar et al., 2006; Cocchiaro et al., 2008; Elwell and Engel, 2012). All down-regulated pathways at 12 h indicate that *Chlamydia*-infected cells are exhibiting stress responses. DNA damage as part of the cell cycle, and repair pathways are enriched, possibly representing a continuation of infection stresses at 6 h, and likely indicative of further *Chlamydia*-induced interruption of the cell cycle. Notably, two p53 associated pathways were enriched from associated genes. p53 expression tightly controls the cell cycle and is modulated in response to activities including cell stress, DNA damage, as well as bacterial infection (Zaika et al., 2015). *Chlamydia-*induced down-regulation of p53 may help to protect infected cells against death-inducing host responses, thus allowing chlamydial survival (Siegl et al., 2014). Only four DE genes from 6 and 12 h overlap. The two up-regulated genes were *DUSP5* (Dual Specificity Phosphatase 5) and *MT1E* (metallothionein 1E), while the two down-regulated genes were *TUBA4A* and *HSP90AA1*.

Comparing cluster 1 (3-h infected cells) against cluster 2 (DE genes from 6 and 12 h) demonstrates a substantial number of DE genes (2,291 up and 3,487 down-regulated) (Figure 6A). Although the model attempted to account for the loss of the 3 h mock-infected cells, we note a proportion of these DE genes may not be related to a productive chlamydial infection as a result. The down-regulated pathways have low combined scores (a combination of *p*-values and z-scores) compared to the up-regulated pathways, which may be explained by the large number of down-regulated non-coding RNAs (ncRNAs), which are typically not incorporated into the underlying enrichment analyses. Three of the up-regulated pathways are associated with infection (*Infectious disease, Influenza life cycle*, and *Influenza infection*; Figure 6B), demonstrating that general infection mechanisms are the key differences between these temporally defined clusters.

## Discussion

To better understand bacterial pathogenesis and resulting disease outcomes, it is critical to understand functional changes to specific cell populations of infected and neighboring cells, and recruited immune cells in the infected tissue context. This is especially relevant for *Chlamydia* which, due to its obligate intracellular niche and distinct morphologies, has long been refractory to research. As a result, many infection and disease processes at the cellular and tissue level remain largely unknown or poorly characterized *in vivo*. Gene expression profiling of *Chlamydia*-infected cells by microarray (Porcella et al., 2015), dual RNA-Seq (Humphrys et al., 2013; Marsh et al., 2018), or other genome-scale analyses (Hayward et al., 2019) are powerful techniques to help deconvolute these interactions and processes. However, these and similar genome-scale analyses of infected cells have typically been performed on bulk cell populations, i.e., infected cell monolayers *in vitro*, or selectively sorted/purified subsets of cell populations. Such bulk cell approaches can potentially miss cell-cell variability, or cells that contribute to overlapping phenotypic characteristics, potentially masking critical biological heterogeneity as irrelevant signals from non-participating cells that can skew the average. This may influence the understanding of multifactorial and dynamic processes, such as inflammation and fibrosis during ascending chlamydial infection. Single cell approaches can potentially alleviate some of these concerns, but also provide new challenges.

We describe the first application of scRNA-Seq to *Chlamydia*-infected cells. This pilot dataset, comprising 264 single infected and mock-infected cells encompassing three early times of the *in vitro* chlamydial developmental cycle, was designed to examine the feasibility and pitfalls of single cell approaches to investigate chlamydial biology and to ultimately identify host-derived transcriptional biomarkers of chlamydial infection. After quality assessment and filtering measures, we retained 200 high quality, *C. trachomatis*-infected and mock-infected cells.

We note that the experimental design used here will not distinguish *Chlamydia*-mediated effects from infection-specific or non-specific epithelial cell responses. In addition, the *in vitro* infections used as the source of the single cells are centrifugation-synchronized in order to minimize the degree of heterogeneity at each infection time to enable more accurate examination of temporal effects. Despite this, lag time of differentiating EBs to RBs between distinct cells may still influence host responses mediated by temporally expressed/secreted chlamydial factors. Given that the minimal chlamydial generation time during exponential growth has been estimated as 2.6–4.6 h (Wilson et al., 2004; Lambden et al., 2006), it is plausible that cells at each time may cluster with an earlier or later time.

Clustering, pseudotime and cell state prediction analyses demonstrated that *Chlamydia*-treated cells at 3 h are readily distinguishable from *Chlamydia*-treated and mock-infected cells at 6 and 12 h. Curiously, cells at 6 and 12 h clustered together and could not be further deconvoluted from each other, possibly showing that host cell transcription at these times is broadly similar. A recent FAIRE-Seq analysis of *Chlamydia*-infected epithelial cells, examining patterns of host cell chromatin accessibility over the developmental cycle (Hayward et al., 2019), found that 12 h post-infection was relatively quiescent in terms of host cell transcriptional activity. This finding is reflected by our scRNA-Seq analyses here and may be extended to 6 h post-infection. In addition, both *Chlamydia*-infected and mock-infected cells at 6 and 12 h clustered together. One interpretation of this phenomenon is that these early infection times represent a period where the ongoing establishment of the inclusion and chlamydial division after initial entry and infection events is largely cryptic to the host cell as manifested by transcriptional processes.

However, limitations inherent to the experimental design and the technology used here may also influence our results, and should inform future single cell experiments. We used an MOI~1, based upon our previous work with bulk dual RNA-Seq (Humphrys et al., 2013), which typically results in highly infected HEp-2 monolayers (95%+) when using *C. trachomatis* serovar E. When combined with the closed nature of the integrated fluidic circuits used to capture individual cells, the early infection times, and the destructive nature of single cell RNA-Seq, using a lower MOI may have led to populations of both infected and uninfected cells to be sampled. In bulk transcriptomic experiments, any distinct signal from a small number of uninfected cells will be largely overwhelmed. In contrast, uninfected cells in single cell experiments may have an outsized effect, particularly if the total number of cells sampled is insufficient. In addition, the population of infecting EBs may include differentially viable EBs, leading to a divergence of transcriptional profiles between cells with productive infections, compared to cells that are initially infected with non-viable EBs that will not proceed to a productive infection. Similarly, any putative “neighbor” effect of uninfected cells next to infected cells may lead to distinct transcriptional profiles. Given the relatively low number of single cells sampled and the early infection times examined, these factors are a potential source of bias in these pilot experiments. With these limitations in mind, it may be more accurate to describe the *Chlamydia*-infected cells in these experiments as “*Chlamydia*-exposed.”

Design of *in vitro* single cell experiments in the *Chlamydia*-infected cell context will benefit firstly from a higher MOI to ensure maximal productive infection. Secondly, collection of much higher numbers of single cells for scRNA-Seq and other single cell genome-scale measurements are now possible, minimizing the potential for introduced sampling biases that arise from low infected cell numbers. Thirdly, the inclusion of additional controls, such as UV-inactivated EBs or opsonized latex beads, will allow host cell transcriptional responses to productive vs. non-productive infection events to be examined, as well as separating host cell infection-specific processes from non-specific phagocytic responses. Finally, moving away from poly(A) capture library construction to random hexamers instead will allow pathogen transcripts to simultaneously be interrogated in the single cell mode, as recently applied to *Salmonella typhimurium-*infected cells (Avital et al., 2017).

A range of cell cycle states were observed in our data. We attempted to remove these effects as potential confounders through bioinformatic means. *Chlamydia*-infected cells are still able to undergo mitosis, however mitosis-related defects do occur during chlamydial infection. These include an increase in supernumerary centrosomes, abnormal spindle poles, and chromosomal segregation defects, and result in a heavily burdened cell that proliferates more slowly (Grieshaber et al., 2006; Knowlton et al., 2011). Cells that recover from infection are still likely to contain chromosome instabilities, which can then be passed down to uninfected daughter cells (Grieshaber et al., 2006). This may be manifested in our data as we see a number of pathways related to the cell cycle that are down-regulated. While this could be an off-target effect of infection that does not benefit *Chlamydia*, interference with the cell cycle may constitute an infection strategy, as *in vivo* cells will be at different cell cycle stages and thus some may be more or less susceptible to infection. Nevertheless, future *in vitro* investigations of chlamydial infection should attempt to explore and/or mitigate these effects through cell cycle arrest strategies (Johnson et al., 2009) prior to infection and/or single cell separation.

Differential expression comparing cells between clusters identified both conserved and temporally specific gene expression over the times examined. Comparison of these differentially expressed genes with other published *Chlamydia*-infected cell transcriptomic datasets (Humphrys et al., 2013; Porcella et al., 2015) showed little overlap (data not shown), most likely as a consequence of an accumulation of technical differences that make direct comparisons difficult, including the relatively small numbers of single cells sampled here, different times post-infection, different MOIs, and different chlamydial serovars and *in vitro* cell lines. Single cell RNA-Seq approaches may also benefit from parallel bulk RNA-Seq approaches from the same input material to cross-check, compare and validate. Nevertheless, many of the identified pathways and genes are directly relevant to known chlamydial infection processes, including metallothioneins, innate immune processes, cytoskeletal components, lipid biosynthesis, and cellular stress. These analyses demonstrate that, despite the limitations of this pilot dataset, distinct host cell transcriptional responses to infection are readily discernible by single cell approaches, even at the early stages of the chlamydial developmental cycle, yielding robust data and confirming that host cell-derived transcriptional biomarkers of chlamydial infection are identifiable. Thus, single cell genome-scale approaches applied to *Chlamydia*-infected and neighboring cells, recruited immune cells from inflammatory processes, and structural cells obtained from clinical swabs or *ex vivo* tissues, are likely to lend significant insight to the complex processes that underpin chlamydial infection and the associated inflammatory disease outcomes.

## Methods

### Cell Culture and Infection

HEp-2 cells (American Type Culture Collection, ATCC No. CCL-23) were grown as monolayers until 90% confluent. Monolayers were infected with *C. trachomatis* serovar E in SPG as previously described (Tan et al., 2009). Additional monolayers were mock-infected with SPG only. The infection was allowed to proceed 48 h prior to EB harvest, as previously described (Tan et al., 2009). *C. trachomatis* EBs and mock-infected cell lysates were subsequently used to infect fresh HEp-2 monolayers. Fresh monolayers were infected with *C. trachomatis* serovar E in 3.5 mL SPG buffer for an MOI ~ 1 as previously described (Tan et al., 2009), using centrifugation to synchronize infections. Infections and subsequent culture were performed in the absence of cycloheximide or DEAE dextran. A matching number of HEp-2 monolayers were also mock-infected and synchronized as above using uninfected cell lysates. Each treatment was incubated at 25°C for 2 h and subsequently washed twice with SPG to remove dead or non-viable EBs. Ten milliliter fresh medium (DMEM + 10% FBS, 25 μg/ml gentamycin, 1.25 μg/ml Fungizone) was added and cell monolayers incubated at 37°C with 5% CO_**2**_. Three biological replicates of infected and mock-infected dishes per time were harvested post-infection into single cell populations by trypsin in sterile PBS prior to immediate single cell capture and library preparation.

### Library Preparation and Sequencing

A Fluidigm C1 instrument was used for cell capture. This instrument uses microfluidics on IFCs to capture single cells, lyse and prepare cDNA, using 96 well-plates as input. Only polyadenylated fragments are captured from each cell, typically restricting analysis to eukaryotic mRNA. Cell lysis, reverse transcription, and cDNA amplification were performed using the C1 Single-Cell Auto Prep IFC. The SMARTer Ultra Low RNA Kit (Clontech) was used for cDNA synthesis and Illumina NGS libraries were constructed using the Nextera XT DNA Sample Prep kit. The resulting 264 single cell libraries were sequenced using the Illumina HiSeq 4000 platform (150 bp paired-end reads) across three batches. Each plate was designed with a balanced distribution of time points and conditions.

### Pre-processing and Quality Control

Raw sequencing reads were demultiplexed using DeML (Renaud et al., 2015) with default settings. Trim Galore (v.0.4.3) (Krueger, 2012) was used to trim adaptors and low quality reads. Confirmation of the removal of adaptors, low quality reads and other quality control measurements was performed with FastQC (v.0.11.5) (Andrews, 2010). Reads were subsequently aligned to the human genome version (GRCh 38.87) with STAR (v.2.5.1a) (Dobin et al., 2013) retaining paired and unpaired mapped reads that were merged in to a single BAM file.

FastQ-Screen (v.0.11.1) (Wingett, 2011) was used to screen for sources of contamination across all cells. This output and low mapping rates confirmed the removal of all 3-h uninfected cells, due to extremely low mapping rates to the Human genome and high mapping rates to other organisms. Features of the remaining cells were counted with FeatureCounts (v.1.5.0-p3) (Liao et al., 2014). MultiQC (v.1.0) (Ewels et al., 2016) was used throughout the previous steps, combining output from each piece of software to easily make comparisons between batches and across time points.

### Identifying Outlier Cells Based on Filtering

Counted features were imported into Scater (v.1.5.11) (McCarthy et al., 2017), where subsequent quality control reduced the total number of cells from 264 to 200. The filter settings were comprised of four steps: (1) total mapped reads should be >1,000,000; (2) total features >5,000; (3) expression from mitochondrial genes <20% of total expression; and (4) expression from rRNA genes comprise <10%.

### Removing Confounding Effects

Cell cycle classification was performed using Cyclone (Lun et al., 2016) prior to filtering out low abundance genes, as recommended. To account for the differences in library sizes between cells, the deconvolution method from Scran (v.1.6.0) (Lun et al., 2016) was used. Further confounding effects such as cell cycle and sequencing batch effects were removed using the RUVs method of RUVSeq (v.1.12.0) (Risso et al., 2014) using *k* = 4. Doublet detection was performed using Scrublet (v.0.1) (Wolock et al., 2019) and DoubletDetection (v.2.4) (Gayoso and Shor, 2019).

### Clustering

Unsupervised clustering was performed using the Single-Cell Consensus Clustering (SC3) package (v.1.10.1) (Kiselev et al., 2017). Two clusters (k) were chosen based on automatic prediction by SC3 after iterating through a range of k (2:10). Higher values of K were examined; however, two clusters remained the best fit to this data as assessed by various internal plots including consensus matrices, silhouette plots and cluster stability plots. To further confirm the two clusters, the KNN-Smoothing (Wagner et al., 2018) (K-nearest neighbor smoothing) function (v.1) was also applied to different transformations of the library normalized data.

### Pseudotime Analysis

TSCAN (v.1.16.0) (Ji and Ji, 2016) was used to perform pseudotime analysis. When all cells were analyzed, the “pseudocount” and “minexpr_value” flags were set to 0.5 in pre-processing to allow more features to be selected, resulting in an increase of cells with assigned cell states, especially when the number of states was manually increased. The default pre-processing settings were used to examine infected cells alone.

### Differential Expression

Most scRNA-Seq differential expression software only allows for direct comparisons, such as cluster comparisons. As our experimental design examined both infected and mock-infected cells and, due to the loss of the 3 h mock cells following QC measures, we used edgeR (v.3.24.3) (Robinson et al., 2010), as it provides better functionality for more complex comparisons than most single-cell specific tools. Initial comparisons were between infected and mock infected cells at 6 and 12 h. The use of edgeR allowed the comparison between 3 h infected cells (cluster 1) and the remaining cells (cluster 2), taking into consideration the differences between infected and mock infected cells from 6 and 12 h. In addition to including the RUVSeq factors of unwanted variation to the model matrix, the dispersion trend was estimated using “locfit” and “mixed.df” flags set to true. Resulting *p*-values were adjusted using a false discovery rate (FDR) < 0.05 and separated based on their respective fold-changes. Significant genes were examined using enrichR (Chen et al., 2013), with enriched pathways from Reactome sorted by their combined scores (a calculation of *p*-values and z-scores) (Croft et al., 2011).

## Data Availability Statement

The data set supporting the results of this article is available in the GEO repository, GSE132525.

## Author Contributions

RH analyzed, interpreted, and co-wrote the manuscript. JM assisted with analysis and interpretation. MH performed the chlamydial infections and scRNA-Seq laboratory methods. WH assisted with interpretation of the data and contributed to the manuscript. GM conceived the experiments, obtained the funding, oversaw the sequencing, data analysis and interpretation, and co-wrote the manuscript.

### Conflict of Interest

The authors declare that the research was conducted in the absence of any commercial or financial relationships that could be construed as a potential conflict of interest.

## Supplementary Material

Supplemental File 1 Demuxing results comparing Illumina and DeML. Demuxing comparison between the standard Illumina software and DeML. 1.8–5.5% additional reads were recovered over each cell batch **(A–C)**.

Supplemental File 2 Biotype distribution. **(A)** Distribution of different gene biotypes by time. **(B)** Total gene expression by biotype. **(C)** Total gene expression by chromosome location.

Supplemental File 3 Contamination of 3-h mock-infected cells. Examples of two 3-h mock-infected cells with unusual mapping to 12 different genomes, indicating cross-contamination. All 3 h mock-infected cells showed similar profiles and were removed from further downstream analysis.

Supplemental File 4 Identifying and removing confounding effects. **(A)** Relative log expression (RLE) plot of gene expression from all cells after removal of confounding effects using RUVSeq. **(B)** Density curve distribution showing variability associated to key variables from raw counts, after library normalization, and after using RUVSeq respectively. **(C)** PCA plot demonstrating two-dimensional structure of cell expression profiles after removing confounding effects. Two variables (Total features and Time-point) account for 99% of the variability at component 1 (PC1).

Supplemental File 5 Doublet detection. **(A)** Scrublet identifies two cells as possible doublets, red bars. **(B)** DoubletDetector found no cells exhibited doublet characteristics.

## Abbreviations

DE: Differentially expressed
EBs: Elementary bodies
FDR: False discovery rate
IFC: Integrated fluidic circuit
IF: Intermediate filament
MT: Metallothionein
ncRNAs: Non-coding RNAs
PID: Pelvic inflammatory disease
ROS: Reactive oxygen species
RLE: Relative log expression
RBs: Reticulate bodies
STI: Sexually transmitted infections
scRNA-Seq: Single cell RNA sequencing
TFI: Tubal factor infertility.
