# Supplementary figures and images for: Early Transcriptional Landscapes of Chlamydia trachomatis-Infected Epithelial Cells at Single Cell Resolution
Source: Front Cell Infect Microbiol. 2019 Nov 19;9:392. doi: 10.3389/fcimb.2019.00392 (PMC6877545; doi:10.3389/fcimb.2019.00392)

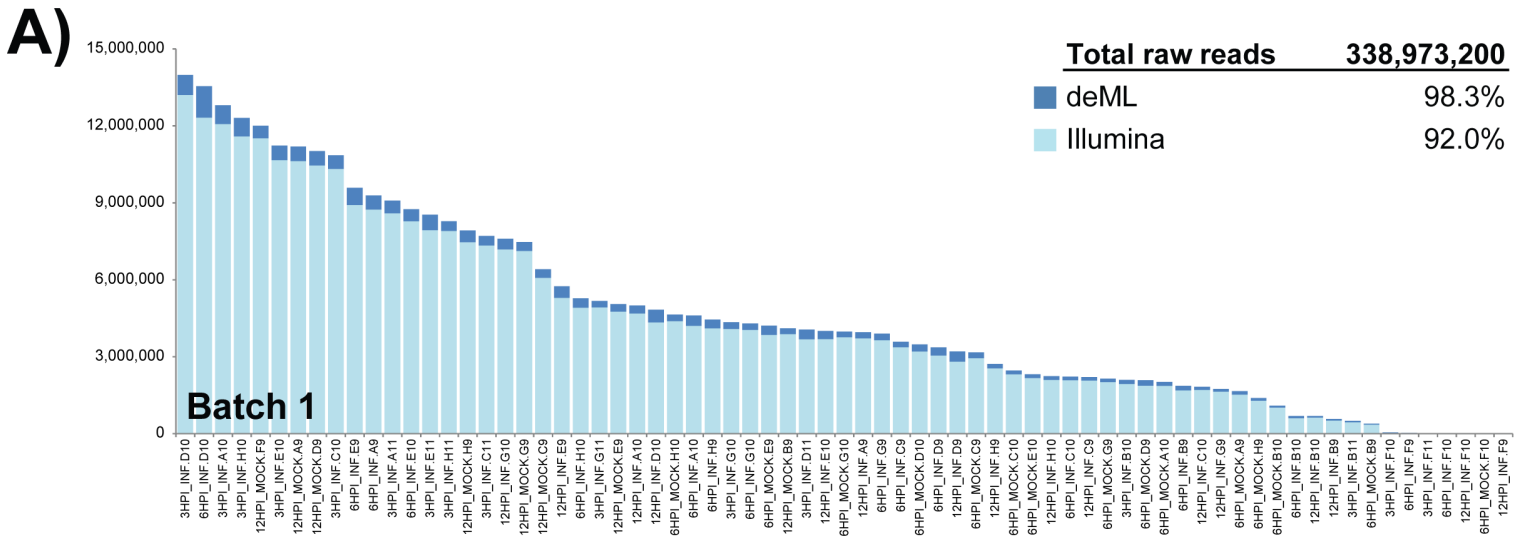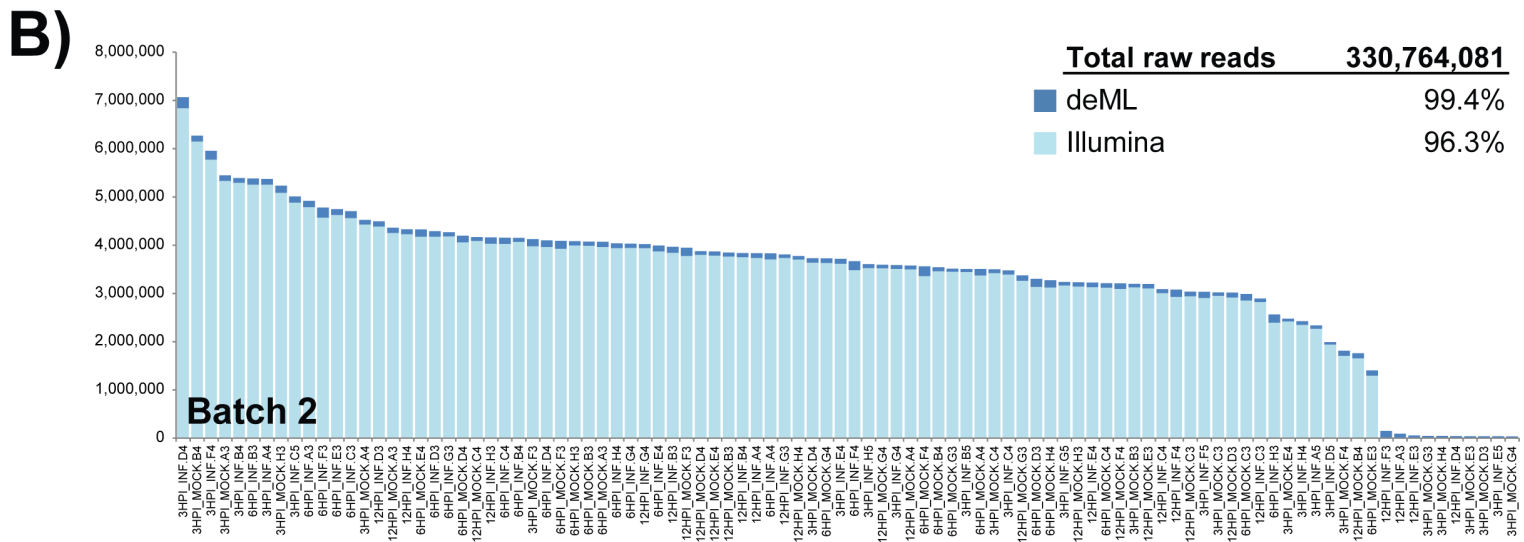

**A) Genetic biotypes**

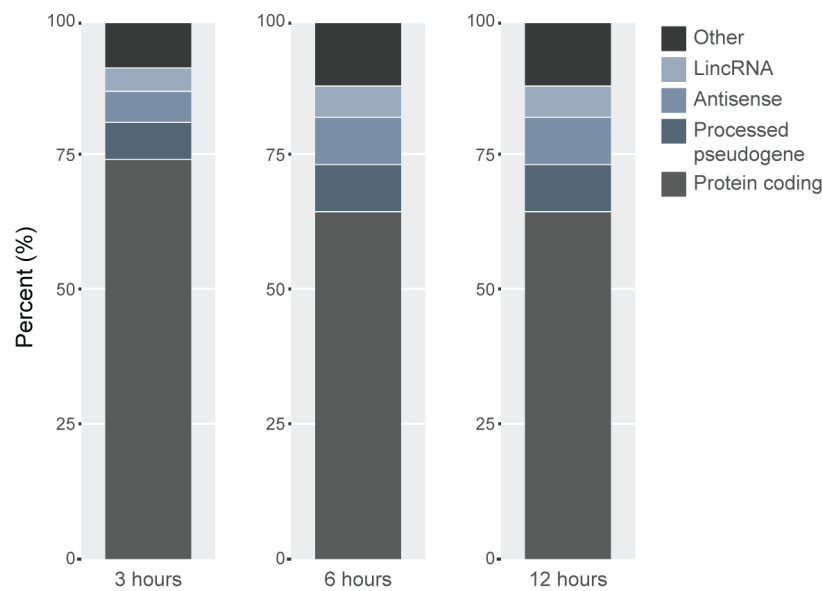

**B) Expression from gene biotypes**

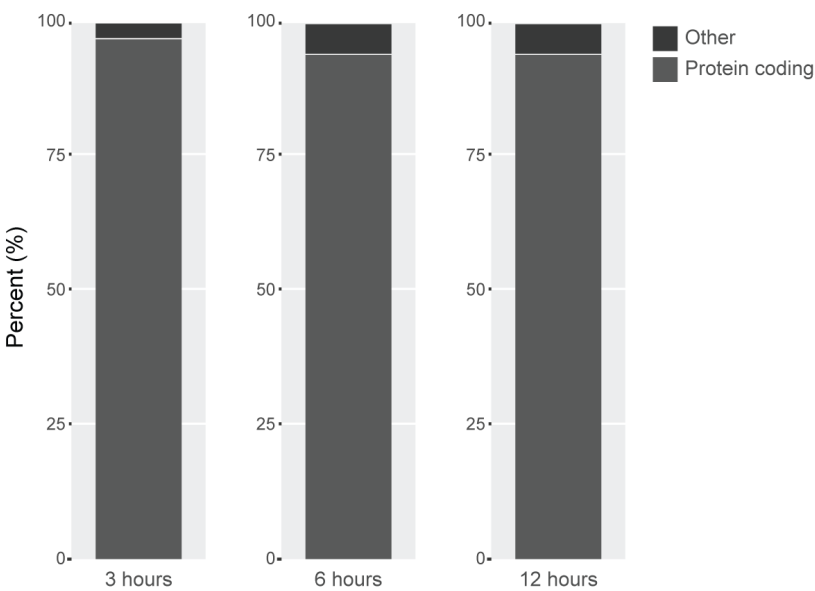

**C) Expression from each chromosome**

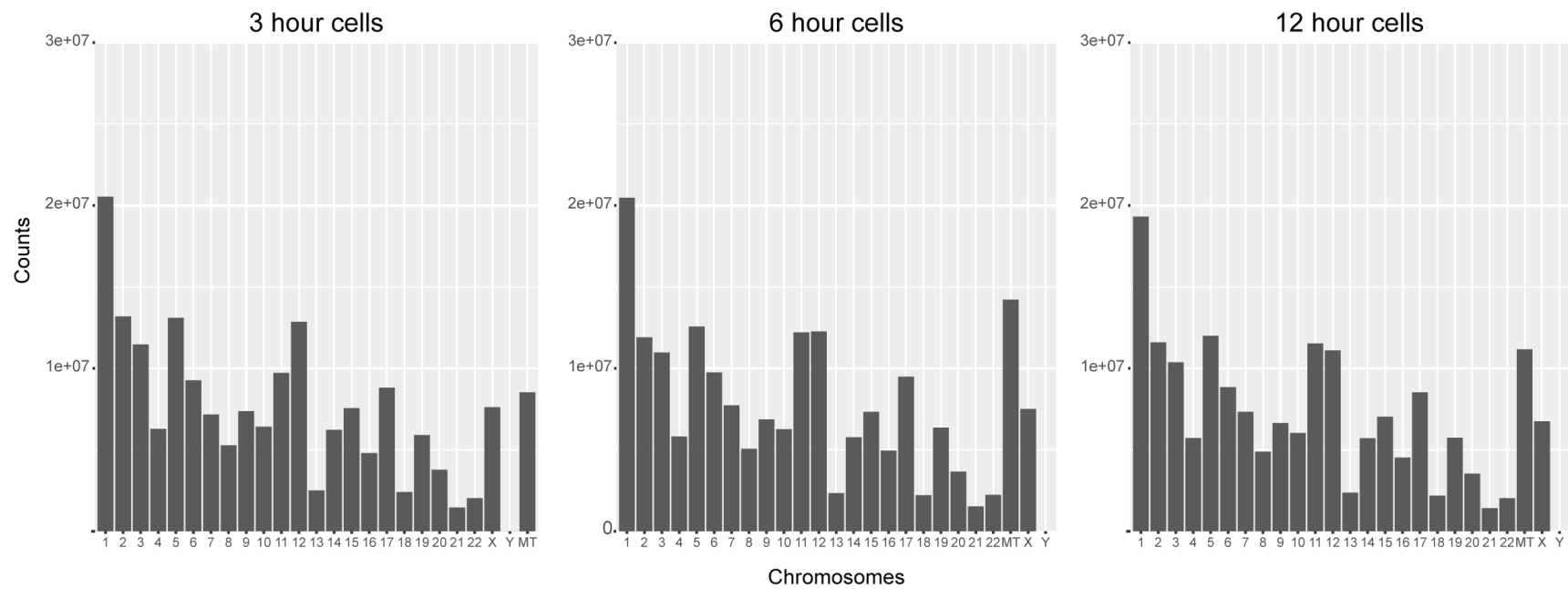

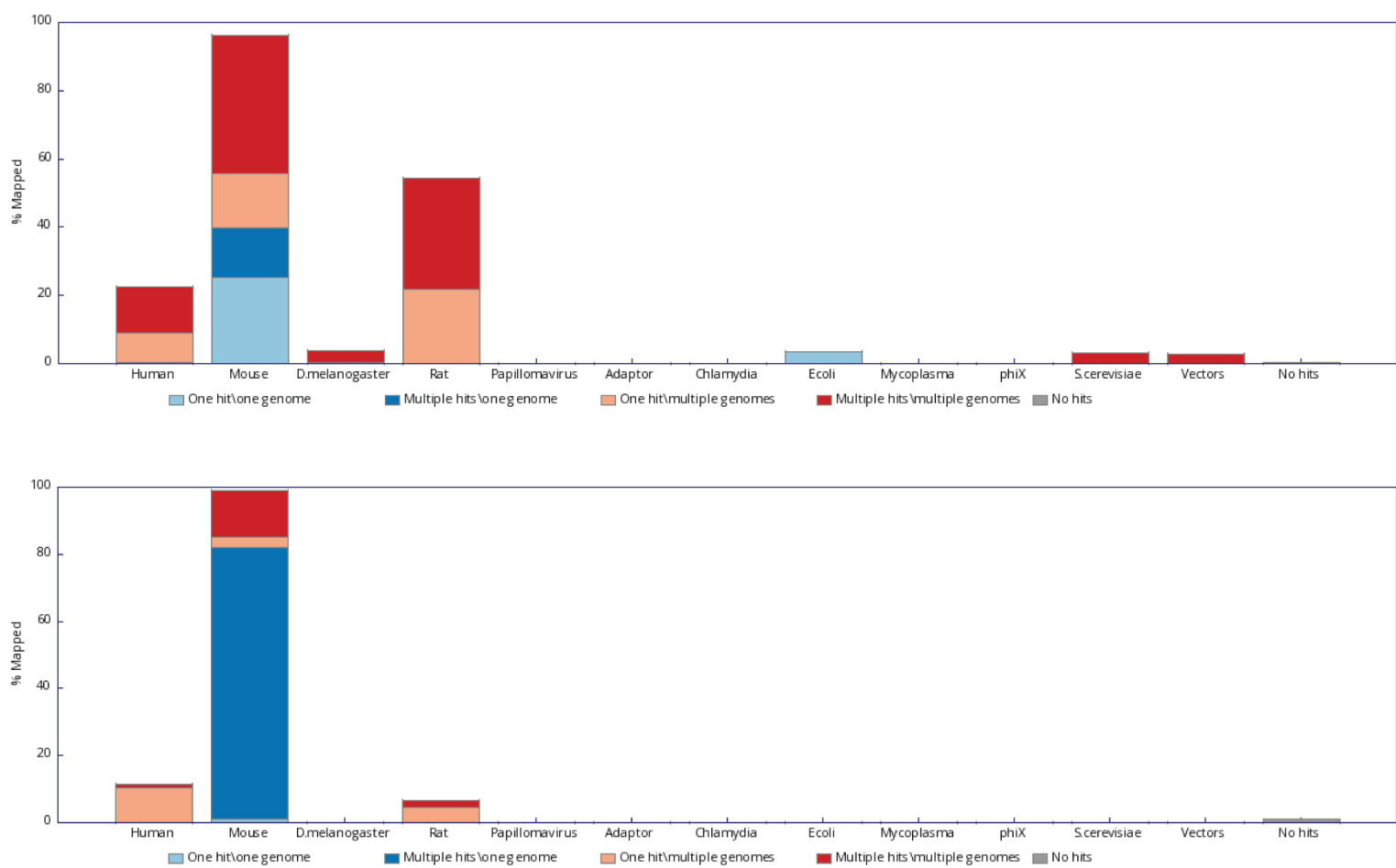

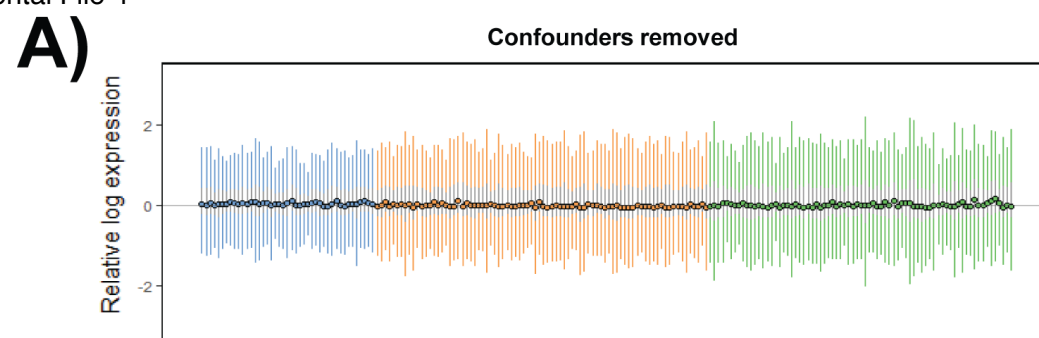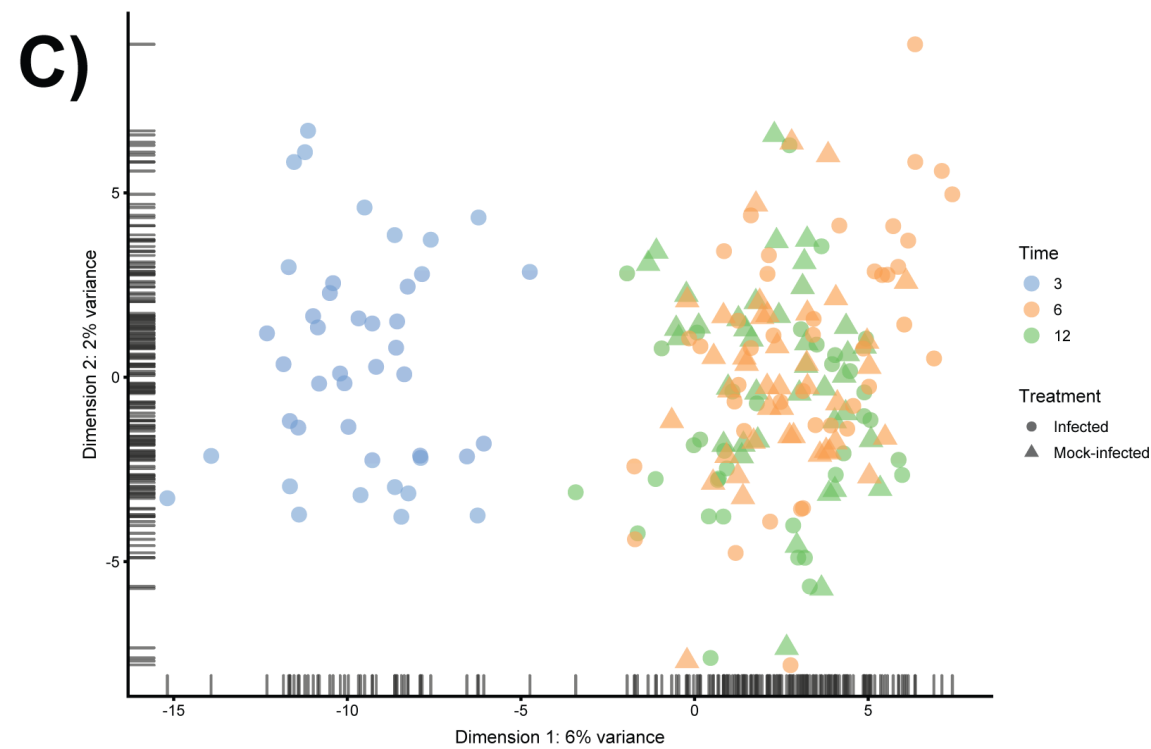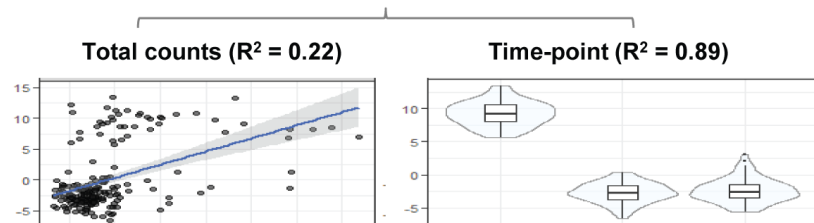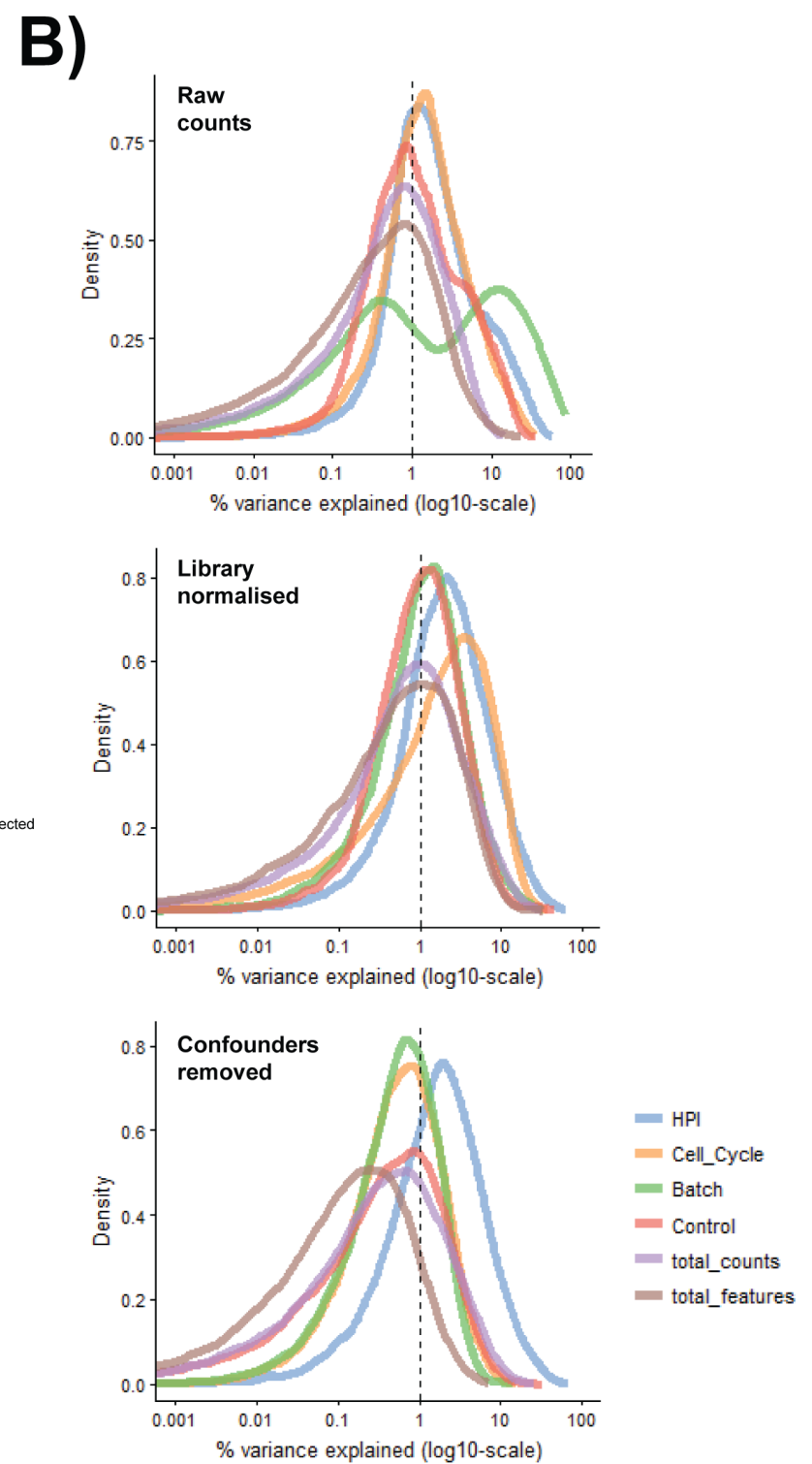

A)

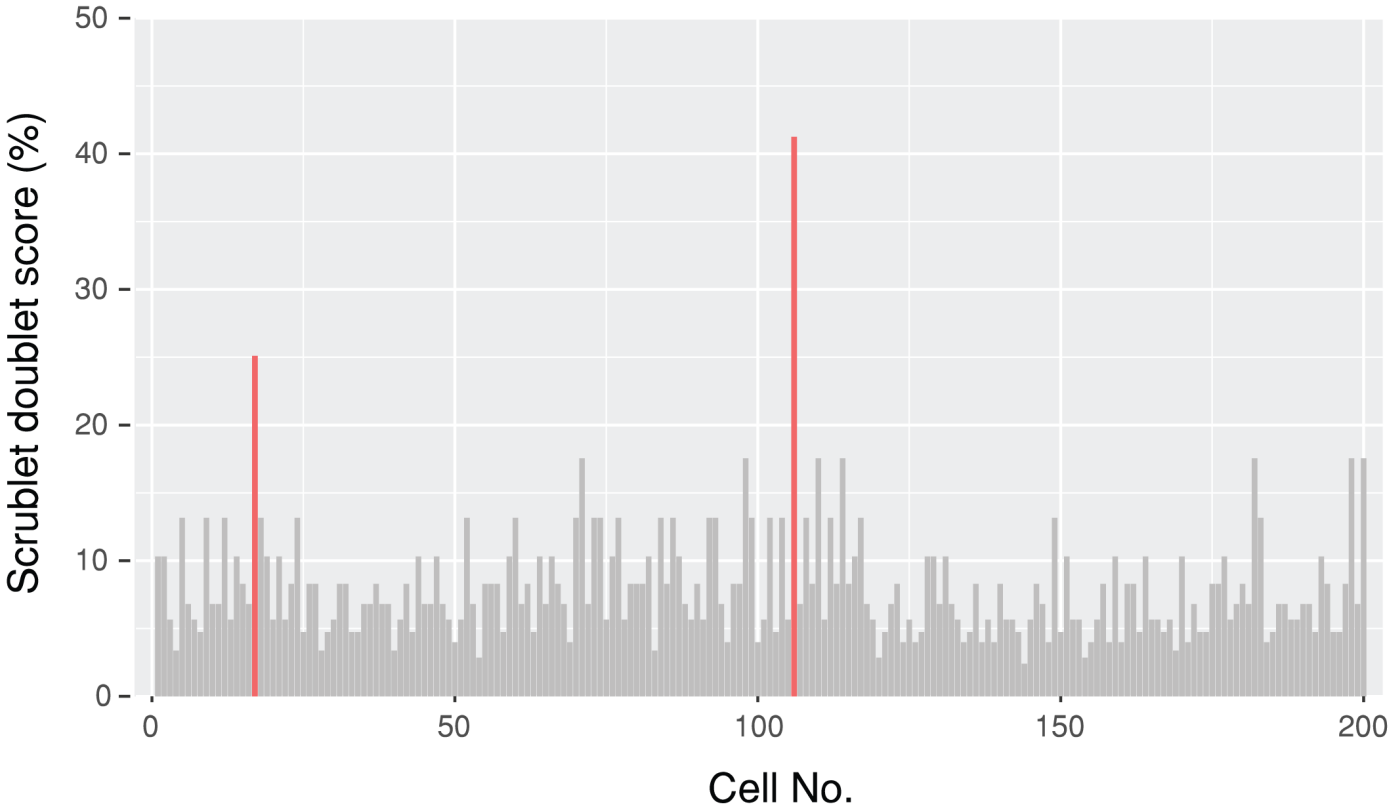

B)

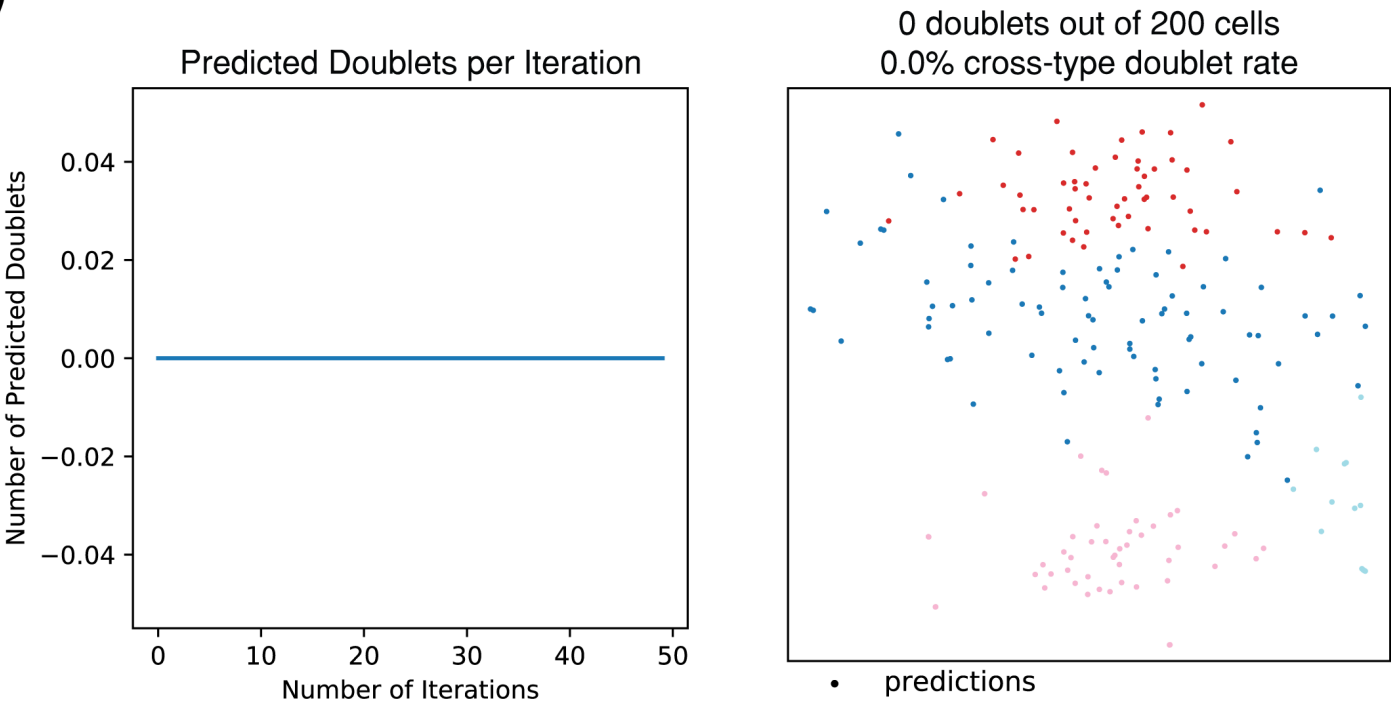

Supplement: Supplemental File 1 — Demuxing results comparing Illumina and DeML. Demuxing comparison between the standard Illumina software and DeML. 1.8–5.5% additional reads were recovered over each cell batch (A–C). [file Data_Sheet_1.pdf]
